# Healthcare utilization, provisioning of post-exposure prophylaxis, and estimation of human rabies burden in Madagascar

**DOI:** 10.1016/j.vaccine.2018.11.011

**Published:** 2018-11-30

**Authors:** Malavika Rajeev, Glenn Edosoa, Chantal Hanitriniaina, Soa Fy Andriamandimby, Helene Guis, Ravo Ramiandrasoa, Rila Ratovoson, Laurence Randrianasolo, Mamitiana Andriamananjara, Jean-Michel Heraud, Laurence Baril, C. Jessica E. Metcalf, Katie Hampson

**Affiliations:** a Department of Ecology and Evolutionary Biology, Princeton University, Princeton, United States; b Service de Lutte contre les Maladies Épidémiques et Négligées, Ministère de la Santé Publique, Antananarivo, Madagascar; c Mention Zoologie et Biodiversité Animale, Faculté des Sciences, Université d’Antananarivo, Antananarivo, Madagascar; d Virology Unit, Institut Pasteur de Madagascar, Antananarivo, Madagascar; e Epidemiology and Clinical Research Unit, Institut Pasteur de Madagascar, Antananarivo, Madagascar; f Vaccination Center, Institut Pasteur de Madagascar, Antananarivo, Madagascar; g CIRAD, UMR ASTRE, Antananarivo, Madagascar; h ASTRE, Univ Montpellier, CIRAD, INRA, Montpellier, France; i Direction des Services Vétérinaires, Ministère chargé de l’Agriculture et de l’Élevage, Antananarivo, Madagascar; j Boyd Orr Centre for Population and Ecosystem Health, Institute of Biodiversity, Animal Health and Comparative Medicine, University of Glasgow, Glasgow, UK

**Keywords:** Disease burden, Contact tracing, Rabies surveillance, Intradermal, Post-exposure treatment, Canine rabies

## Abstract

In Madagascar, dog-mediated rabies has been endemic for over a century, however there is little data on its incidence or impact. We collected data over a 16-month period on provisioning of post-exposure prophylaxis (PEP) at a focal clinic in the Moramanga District and determined the rabies status of biting animals using clinical and laboratory diagnosis. We find that animal rabies cases are widespread, and clinicbased triage and investigation are effective ways to increase detection of rabies exposures and to rule out non-cases. A high proportion of rabies-exposed persons from Moramanga sought (84%) and completed PEP (90% of those that initiated PEP), likely reflecting the access and free provisioning of PEP in the district. Current clinic vial sharing practices demonstrate the potential for intradermal administration of PEP in endemic African settings, reducing vaccine use by 50% in comparison to intramuscular administration. A high proportion of PEP demand was attributed to rabies cases, with approximately 20% of PEP administered to probable rabies exposures and an additional 20% to low-to-no risk contacts with confirmed/probable animal or human cases. Using a simplified decision tree and our data on rabies exposure status and health-seeking behavior, we estimated an annual incidence of 42-110 rabies exposures and 1-3 deaths per 100,000 persons annually. Extrapolating to Madagascar, we estimate an annual burden of 282-745 human rabies deaths with current PEP provisioning averting 1499-3958 deaths each year. Data from other clinics and districts are needed to improve these estimates, particularly given that PEP availability is currently limited to only 31 clinics in the country. A combined strategy of mass dog vaccination, enhanced surveillance, and expanded access to PEP along with more judicious guidelines for administration could effectively reduce and eventually eliminate the burden of rabies in Madagascar.

## 1 Introduction

To date, canine rabies is estimated to cause around 60,000 human deaths annually [1]. Infection is completely preventable if exposed individuals receive prompt post-exposure prophylaxis (PEP), but most human cases occur in low-income countries in Africa and Asia, where access to PEP is often limited [2]. Mass dog vaccination has proved effective in preventing human rabies in many countries [3]. The WHO and their partners have set a target for an end to human deaths due to dog-mediated rabies by the year 2030 [4]. This goal will require delivering vaccine interventions in both domestic dog and human populations in resource-limited settings where canine rabies remains endemic.

In Madagascar, the first human rabies case was reported in 1896. The Institut Pasteur de Madagascar (IPM) has provided PEP free-of-charge to patients in the country since 1902 [5]. Until 2006, IPM provided rabies nerve-tissue vaccines to all district health centers. In 2006, use of nerve tissue was discontinued in the country, and shifted to use of the Purified Vero Cell Rabies Vaccine solely, but with provisioning to only a subset of district health centers [6]. Currently, there are 31 anti-rabies medical centers (ARMC, also referred to as Centre de Traitement Antirabique) across the country. Each ARMC is located in a public hospital or health care center, and there are no other sources of PEP available publicly or privately in the country. At the time of the study, all ARMC were using the modified Thai Red Cross (TRC) protocol (i.e. 2 intradermal injections of 0.1 mL at two sites, deltoids and/or thighs, on days 0, 3, 7 and 28). Purified equine rabies immunoglobulin (RIG) is only available at the IPM ARMC in the capital city Antananarivo, and patients from peripheral ARMC are supposed to be referred to the capital when RIG is necessary. Both vaccine and RIG are administered free-of-charge to patients. Culling is the official policy to respond to any suspected or confirmed animal rabies case (Decret No. 95-375,1995) and dog vaccines are limited in availability and only at a high cost to owners.

As rabies control in the dog population is minimal and *ad hoc*, and PEP availability is limited to the 31 ARMCs, there is likely a significant burden of rabies in Madagascar. The National Rabies Reference Laboratory (NRRL) at IPM in Antananarivo is the only facility with the capacity for rabies diagnostic testing [5] and most of the samples submitted to the laboratory come from the capital city and surrounding peri-urban areas. Even with this limited surveillance, 62% of submitted animal samples tested positive for rabies between 2010 and 2015 [7], with cases of canine rabies having been recorded in 38 out of the 114 districts in the country [6]. Annually, between 4 and 10 human rabies cases and 21-111 animal rabies cases are laboratory confirmed [7], but underreporting of both human and animal cases is likely substantial.

To better understand the burden of rabies in Madagascar and PEP functioning in this context, we collected data on reported animal bites and vaccine provisioning at the ARMC in the Moramanga District over a 16-month period (Sep 2016-Dec 2017). We followed up on bite patients to assess the rabies status of biting animals through clinical and laboratory diagnosis and contact tracing to identify unreported exposures. Using the resulting estimates of health seeking behavior, PEP provisioning and adherence, and the incidence of rabies exposures, we applied a simplified decision tree to estimate the number of deaths averted and the current burden of human rabies in the Moramanga District and extrapolate this across Madagascar.

## 2 Methods

### 2.1 Study site

The Moramanga District is located mid-way between the central highlands and the east coast of Madagascar, at an average altitude of 936 m. It comprises 21 communes, covering approximately 7150 km^2^ with a human population between 300,000–350,000 people (www.worldpop.org, [8]). The Moramanga ARMC is located in the Emergency Room (ER) of the District Hospital. The clinic uses 0.5 mL vials of Verorab (Sanofi Pasteur) anti-rabies human vaccine provided through IPM. Generally, if a vial is opened, it is used within one working day. The ER staff requests that patients report between 9 AM–2 PM for PEP to facilitate vial sharing, with one vial split between two patients. Whether animal bites are treated as emergencies and given PEP after these hours depends on the on-call physician. Patients first reporting to other public or private hospitals depend on clinician referral to report to the ARMC. The nearest other health facility offering PEP is the IPM ARMC in Antananarivo, which is a minimum of 2–3 h travel time from anywhere in the district. Patients requiring RIG are referred to IPM.

### 2.2 Data and analyses

From September 2016 to December 2017, we collected baseline data from clinic registers on PEP administration and patient throughput, including patient demographics. The clinic does not track vial use, so we calculated a conservative minimum estimate of vaccine waste (i.e. not accounting for errors in administration, breakage etc.), based on incomplete vial sharing. Current practice is for a single 0.5 mL vial to be split between two patients, with each receiving 0.2 mL of vaccine (2 × 0.1 mL injections/visit), and the remaining 0.1 mL of vaccine considered wastage. We assumed a further 0.2 mL is wasted on each day with an odd number of patient presentations (for vials used only by one patient, with the remaining 0.3 mL discarded). We also calculated the minimum estimate of vials required and wastage under alternative scenarios: with 5 × 0.1 mL injections obtained per 0.5 mL vial; adopting the newly recommended abridged 1-week ID regimen administered on day 0, 3, and 7; and adopting these two practices in combination. We compared these scenarios to the minimum number of vials necessary given intramuscular (IM) administration (Essen 4-dose or Zagreb) with one vial used per injection and assuming no change in compliance.

For all patients, the clinic collects data on the biting animal species and compliance to PEP, but not on which patients are referred and/or receive RIG. We used data from patients who were either bitten or resided in Moramanga that reported to the IPM ARMC during this period to identify those that received RIG, although we did not have data for how many were referred in total. For a subset of biting animals, samples were collected either by local veterinary surveillance officers submitting the whole head or animal for testing) or using the straw method for brain tissue collection [9]. Samples were sent to the NRRL at IPM for diagnostic confirmation using the fluorescent antibody test (FAT). Probable human rabies cases were identified from patients who presented to the clinic with neurological signs, had a history of an animal bite, and died shortly after, or deaths reported to the district health office also with consistent history. One suspect human case was confirmed (positive RT-PCR result on a skin biopsy from the neck [10]).

We interviewed as many patients as possible to classify their rabies exposure status. We classified animals and people (in terms of their exposure to rabies), according to the following *case definitions:*

- **Confirmed case/exposure**: an animal or human that tested positive (by FAT or RT-PCR, respectively) for rabies/a person bitten or scratched by an animal that tested positive for rabies.
- **Probable case/exposure:** an animal or human that was classified as probable for rabies/a person who was bitten or scratched by an animal that was classified as probable for rabies based on adapting the six-step method, with probable cases defined as showing at least one clinical sign and dying or disappearing within 10 days of the bite [11].
- **Contact with a confirmed/probable rabies case:** any person in contact with a confirmed/probable human or animal rabies case as defined by the current national guidelines for ARMCs:
  - Touching the mouth or saliva, or sharing food and drink (human case).
  - Manipulating the body or helping to bury (human case).
  - Licks or contact with the saliva 15 days preceding death (human or animal case).
  - Taking a sample from a suspect animal or human case.

For the majority of these patients, the type of contact was not recorded at the clinic.

- **Non-case/non-exposure:** an animal that was determined to not be rabid/ a person who was bitten or scratched by an animal that was determined to not be rabid (i.e. an animal that remained alive 10 days after the bite or tested negative).
- **Unknown case/exposure:** an animal/patient for whom we were unable to assign a status to, either due to ambiguity in the case history or who, despite attempts via phone call or household visit, we were unable to locate.

For patients reporting between Sep 2016-July 2017, we verified the status of the animal retrospectively through phone interviews or household visits. From August 2017 until December 2017, we interviewed patients directly at the clinic and triaged patients for follow-up, only conducting household visits for cases assigned as probable rabies or for those we were unable to interview at the clinic. Household visits were also limited by accessibility due to road conditions.

**Fig. 1:**
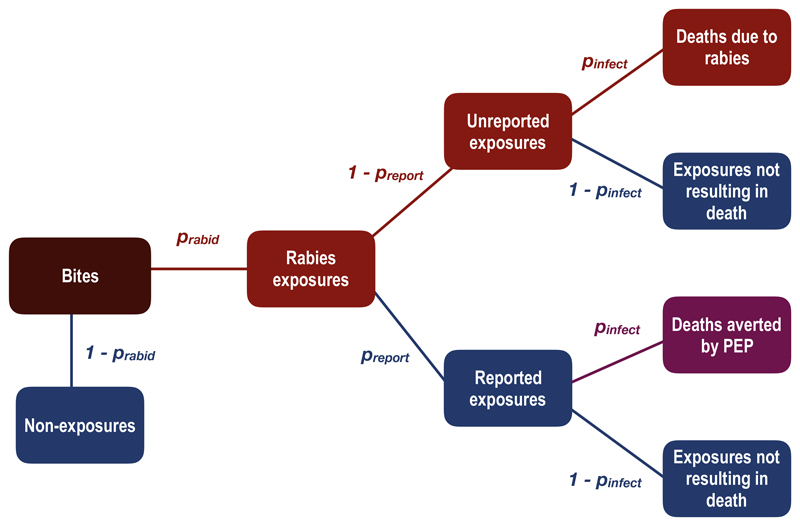
Adapted decision tree framework to estimate burden of human rabies deaths and deaths averted by PEP. We considered that some proportion of total bites in the population (expected bites annually, dark red box) are genuine rabies exposures (Bites × prabid = Rabies exposures), and non-exposures ((1 - prabid) × Bites) do not contribute to rabies deaths or averted deaths. Of the genuine rabies exposures, a fraction present to an ARMC and all of these persons receive PEP (Rabies exposures × preport = Reported exposures). Some of these exposed persons would otherwise have become infected and died if they had not received PEP (Reported exposures × pinfect = Deaths averted by PEP). Of the unreported exposures, a proportion will die due to rabies infection (Unreported exposures × pinfect = Deaths due to rabies). (For interpretation of the references to colour in this figure legend, the reader is referred to the web version of this article.)

We adapted established decision tree frameworks [1,12] to estimate the burden of human rabies and deaths averted through use of PEP using parameters derived from these data. We assumed that no deaths resulted from incomplete or delayed PEP, including the absence of RIG, and that all people who report to an ARMC receive PEP, i.e. no shortages or vaccine refusal. We excluded contacts with (rather than exposures to - see case definitions above) confirmed/probable cases from this analysis, as these pose minimal to no risk of infection [13]. We calculated human rabies deaths and deaths averted by PEP according to the decision tree described in Fig. 1, where *p_rabid_* is the proportion of reported bites that are considered rabies exposures (due to confirmed/probable rabid animals), *p_report_* is the proportion of rabies exposures that present to an ARMC, and *p_infect_* is the probability of infection, and thus death, given a rabies exposure [14]. We calculated an annual bite incidence in the district by taking the average monthly bites per 100,000 recorded in our data during the period of systematic triage (Aug 2017-Dec 2017) and multiplying by 12. Population estimates were taken as the midpoint between 2015 and 2020 UN adjusted population projections from World Pop (www.worldpop.org, [8]). All data were collected using tablets (Samsung Tab 4 and Tab A) using forms from the Wise Monkey Portal ((http://www.wisemonkeyfoundation.org/) and the Device Magic application (https://www.devicemagic.com), and associated data were submitted and stored in secure cloud-based servers. All data analysis and figures were done in R (version 3.5.0, R Core Team, 2018).

### 2.3 Ethics statement

This research was approved by the Princeton University IRB (# 7801) and the Ministry of Public Health Ethics Committee (# 105-MSANP/CE). Oral informed consent was obtained from all interviewed participants. Sample collection from animal carcasses was approved through the Princeton University Institutional Biosafety Committee (# 1105-16) and the Animal Use and Care Committee (# 2079A-16).

## 3 Results

### 3.1 PEP provisioning at the clinic

**Fig. 2:**
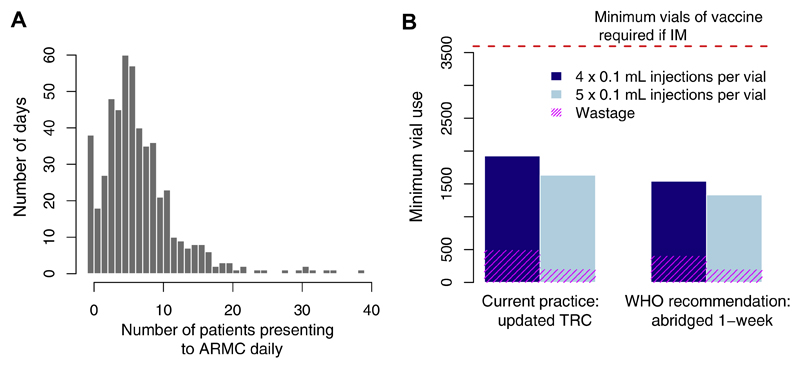
PEP administration and vaccine use. (A) Distribution of observed daily patient presentations (i.e. the number of days with N patients reporting to the ARMC) and (B) calculation of the minimum volume of vaccine (mL) used under current practice with PEP administered according to the updated TRC regimen or according to the latest WHO recommendations with the abridged 1-week ID regimen. Use of 4 × 0.1 mL per 0.5 mL vial (current practice) vs. 5 × 0.1 mL injections per 0.5 mL vial were also compared. The red dashed line corresponds to vaccine use under IM administration, assuming 1 vial per IM injection and the same level of compliance (i.e. under the Essen 4-dose or Zagreb regimen). (For interpretation of the references to colour in this figure legend, the reader is referred to the web version of this article.)

Between September 2016-December 2017, a total of 1019 patients reported to the ARMC. Multiple patients were likely to present on a given day, with only 3% of days where a single patient reported. On average, 7 patients presented per day, but this distribution was skewed with 10 or more patients reporting on 22% of days, and zero patients on only 7% of days (Fig. 2A). Using the updated TRC regimen, an estimated 1927 vials (of 0.5 mL) were required over the study period given the observed daily throughput of ARMC patients. Current ID administration requires approximately 50% less vaccine vials compared to an IM regimen (3597 vials) (Fig. 2B). Use of the abridged 1-week ID regimen could reduce vial use by 20% and drawing 5 × 0.1 mL injections per vial rather than 4 would further reduce vial use by up to 31%. In general, extracting 5 × 0.1 mL injections from a vial reduces the volume of vaccine wasted by 40–50% (Fig. 2B).

### 3.2 Rabies status and characteristics of biting animals

**Fig. 3:**
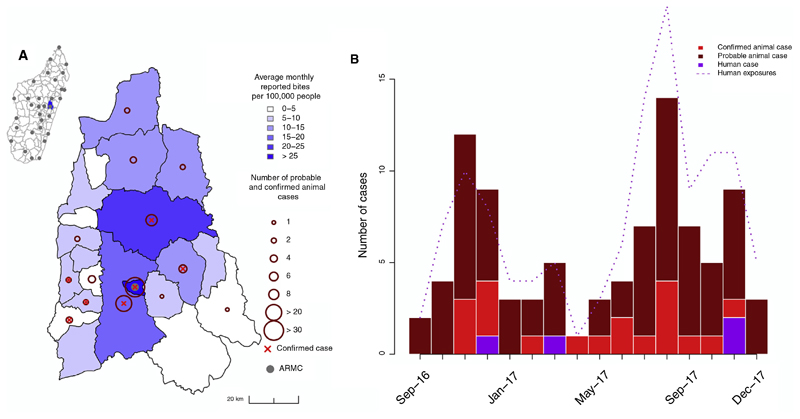
Rabies in the Moramanga District. (A) Average monthly reported bite incidence (blue shading) per commune and total numbers of probable or confirmed cases (dark red circles). A red × indicates if at least one animal case was confirmed in the commune. All coordinates are the commune centroid, and the inset shows the district (in blue) in relation to the other districts (polygons) and ARMC (grey points) in Madagascar. (B) Time series of probable and confirmed animal cases and human cases (bars), as well as total confirmed/probable rabies exposures (dashed line) from September 2016 to December 2017. (For interpretation of the references to colour in this figure legend, the reader is referred to the web version of this article.)

Of the 704 biting animals that were identified at the clinic and through contact tracing, domestic dogs made up the majority (87.5%), followed by cats (9%). Other species (<4% of biting animals) included cows, rodents, one lemur, and one bat. The majority were owned animals (56.7%). We followed up on 390/704 of these animals and identified that 67 were probable cases and 19 were confirmed cases, responsible respectively for 88 probable and 32 confirmed human exposures (Table 1, Fig. 3). Almost all of these confirmed/probable rabid animals were domestic dogs (76/87). Rabies was widespread, with confirmed/probable cases detected in 14/21 communes in the Moramanga District (Fig. 3A). In addition, there was at least one confirmed case in 11/16 months and at least one probable or confirmed case detected in each month of the study. There were also 4 human cases (1 confirmed, 3 probable) reported in the district during this period (Fig. 3B).

Amongst probable and confirmed animal rabies cases, unprovoked aggression was the most common clinical sign followed by excessive salivation; other clinical signs were observed less frequently (<15% of probable/confirmed cases). Confirmed and probable animal cases were more frequently involved in biting multiple people (42.5%) than non-cases (5.4%). They also more frequently bit several animals. In contrast, provoked bites were twice as common amongst non-cases (Table 1). Generally, clinical signs were noted for both non-cases and probable cases, and bites from a probable animal could also be classified as provoked based on our criteria (so could not be used to rule out rabies). A source of infection was only identified for nine confirmed or probable cases (i.e. either an observed bite or signs of a bite prior to biting or the onset of clinical signs). Owners or community members rarely observed when rabid animals bit other animals.

Most probable or confirmed rabid animals were killed after biting or attempting to bite people or other animals (42.5%), or died from disease (26.4%) or other causes (10.3%, including being hit by car, poisoned, or dying from injuries). The remaining 18.6% disappeared after the bite. The majority of animals classified as non-cases were alive 10 days after the bite (94.4%), but three animals that were not alive within 10 days of the bite subsequently tested negative, and eight died at a later date (>10 days after the bite, Table 1).

Of the animals we investigated, 20.5% were reported to have been placed in veterinary observation. Seven of these 80 observed animals were considered to be probable rabies cases by the veterinarian. However, we did encounter two cases where the veterinary conclusion differed from our case determination (one probable case that was declared a non-case by the veterinary officer due to the age of the animal, i.e. <3 months, and the other that was alive at the time of our investigation approximately 3 months after the bite case, which was declared a suspected rabies case by the veterinary officer at the time of the visit). 17% of the animals we investigated were reported to be vaccinated, with 29.2% of non-cases and 28.7% of those placed in veterinary observation reported to be vaccinated. No probable or confirmed animals had a history of vaccination.

### 3.3 Exposure status, health-seeking behavior, and PEP compliance of bite victims and patients reporting to the ARMC

Of the 1019 patients presenting to Moramanga AMRC, 1.5% were in transit and only completed a subset of doses at the clinic. A further 6.8% came from outside of the district but completed their PEP course in Moramanga; these mostly came from the neighboring district of Anosibe An’Ala (41/63), which does not have an ARMC and is a minimum of 12 h travel time from the Moramanga ARMC. Twelve patients were bitten outside the District, but resided in Moramanga and completed their PEP course at the ARMC. We excluded patients bitten in other districts from further analyses.

Excluding contacts with a confirmed/probable case (N = 197), we were able to classify the status of 41.1% of human exposures over this 16 month period, however this proportion varied over time. By conducting clinic-based triage, we were able to classify double the proportion of bite patients to a known exposure status (27.4% of patients pre-August 2017 vs. 61.4% post-August 2017, Fig. 4A). Of the 399 patients we followed up with, we were unable to assign an exposure status to 25.8% (i.e. ‘Unknown’).

Reporting delays were on average 2.8 days for probable exposures and 1.5 days for confirmed exposures, with 61.1% of patients reporting within 2 days of the exposure overall (Table 2). Overall PEP completion was high, with 89.7% of patients completing at least 3 doses (88.1% of probable or confirmed rabies exposures). Approximately 1.7% of patients completed more than 4 doses, as clinic protocol was to restart the course if there was a delay between PEP doses. Eighteen patients from the Moramanga District were recorded at IPM, and fourteen of these patients received RIG (4 confirmed, 3 unknown, and 7 non-exposures).

**Fig. 4:**
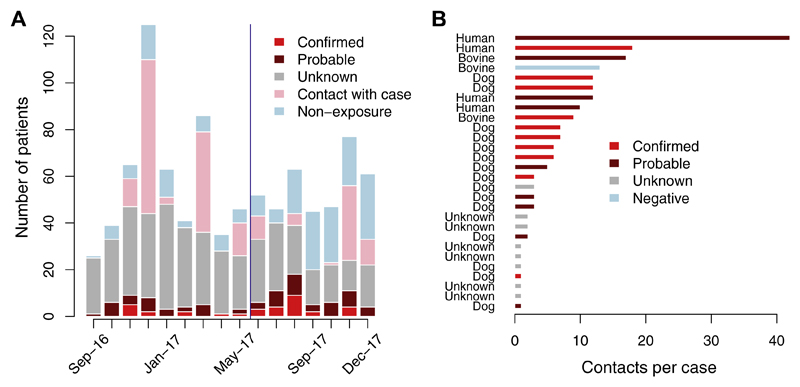
Patients reporting to the ARMC. (A) Monthly time series of patients reporting to the ARMC by their exposure status; the blue line indicates when systematic triaging of patients at the clinic began. (B) Number of contacts per probable case and the rabies status of the case (one bovine case tested negative after sample submission). (For interpretation of the references to colour in this figure legend, the reader is referred to the web version of this article.)

A total of 201 patients reported as contacts with a probable rabies case (human or animal), making up 20% of patients receiving PEP. Fig. 4B shows the distributions of contacts per case, with contacts with the four human cases comprising the majority of these patients (41.6%). One bovine case, for which the contacts were people that consumed the meat of the animal, subsequently tested negative. In addition, details about the nature and timing of the contact, and for a subset details on the probable animal itself (6 unknown cases), were not recorded at the clinic.

Overall, demographics of patients were skewed male (59.1%) and 15 years of age or younger (39.9%), with almost 50% of probable/confirmed rabies exposures 15 years of age or younger compared to 37% of the population in that age group in Moramanga (R. Ratovoson, unpublished data from the Health and Demographic Surveillance System (HDSS) in 3 communes of Moramanga district). For interviewed bite victims (N = 399), we also had data on characteristics of the exposure and post-bite response. The majority of wounds were superficial, but with skin broken. Bites to the head or neck and non-superficial bite injuries made up a small fraction of overall exposures (~9%), and most wounds were reported to be from bites vs. scratches. Overall, 81.4% of interviewed bite victims reported washing the wound with soap and water (Table 2). 13% of interviewees reported to peripheral clinics before reporting to the ARMC, with 42 of these 52 patients reporting to a peripheral clinic before reporting for PEP at the ARMC and the 10 remaining patients reporting only to a peripheral clinic (i.e. did not report for PEP).

We identified a total of 27 people that did not seek PEP, 19 of which were confirmed or probable rabies exposures, resulting in four human deaths (details in Table 3). The remaining 23 were identified during contact tracing investigations and were in good health at the time of investigation; nine of these people reported for PEP after the investigation (5 probable, 2 confirmed exposures, 2 non-exposures). Of the 17 people that reported a reason for not seeking PEP, most were due to ignorance/misconceptions about rabies (n = 9, including thinking the animal was too young to be infected with rabies, not thinking a scratch could result in transmission, reliance on traditional medicine, and complete ignorance of PEP/rabies) and lack of funds to travel to the health center (n = 8).

### 3.4 Deaths averted and current burden of human rabies

We calculated an overall incidence of 189 bites per 100,000 people annually. Given this bite incidence and other parameters (Table 4), we estimate between 19 and 50 deaths averted by PEP and between 4 and 9 human rabies deaths in the Moramanga district annually. Extrapolating to the population of Madagascar, we estimate a current burden of 282–745 human rabies deaths annually, with PEP averting an additional 1499–3958 human rabies deaths. Overall, we estimate a rabies exposure incidence of 42–110 per 100,000 persons annually.

## 4 Discussion

### 4.1 Key findings

Our results demonstrate that canine rabies is widespread in the Moramanga District and results in a high incidence of human exposures. Current free provisioning of PEP to patients is estimated to prevent the majority of deaths resulting from these exposures. Furthermore, clinic practice of ID administration of PEP uses half the vaccine volume compared to IM administration and shows how vial sharing practices can be implemented effectively in an endemic setting in sub-Saharan Africa. Despite these successful practices, canine rabies is still responsible for a significant burden of human deaths and drives high demand for PEP. The substantial costs of procuring and provising free PEP are currently borne by IPM, but would otherwise fall to Madagascar’s health system and/or patients potentially leading to more human rabies deaths.

While approximately 20% of patients reporting for PEP were classified as rabies exposures, an additional 20% were due to low-to-no risk contacts with confirmed or probable animal and human cases, many of which do not fit the WHO case definition for a rabies exposure [13]. Vaccination of loosely defined exposures has been reported in Bhutan, as well, where PEP is provided at no-cost to patients [15]. These practices may jeopardize vaccination of at-risk persons when PEP availability is limited, as occurred during March 2018 when limited vaccine stocks were used to vaccinate 42 contacts around a human case and subsequently resulted in a stockout at the Moramanga ARMC. Training to ensure that health workers can effectively obtain 5 × 0.1 mL injections from 0.5 mL vaccine vials would also enable more people to be treated with potential to reduce costs and the risk of vaccine shortages. Rabies control in the dog population would reduce the number of rabies exposures and contacts with human and animal rabies cases, and could therefore reduce the demand for PEP by over 40%.

Six times as many animal cases were laboratory confirmed during our study period than in the previous 16 months (three animal cases confirmed in the district). Given that between 2011 and 2015, an annual average of 60 rabies case were confirmed in the country, our results suggest significant underreporting of animal rabies both in the Moramanga district and nationally. Through combined clinical and laboratory diagnosis, we were able to determine the rabies status of ~40% of biting animals overall and a higher proportion of animals investigated (~70%). Clinic-based triage of bite patients doubled the proportion of exposures we were able to classify. While we did not detect many linked animal cases (either source or secondary) through contact tracing as demonstrated previously in Tanzania [16], we identified an additional 19 probable/confirmed exposures, 7 of whom reported to the ARMC after the investigation. In Madagascar, when investigations of suspected human and laboratory confirmed animal cases are conducted leads to the provision of PEP to people who have been in contact with these animals or people. However, shifting effort from these case investigations which focus on vaccinating loosely-defined and likely low-risk contacts, towards routine investigations of probable rabid animal bites to identify untreated exposures, could be a more effective response to prevent human deaths while increasing case detection as part of surveillance [17,18].

### 4.2 Strengths and limitations

We did not address the potential misclassification of rabies cases through our investigations. Overall, 86% of samples from suspected animals were confirmed positive; however, only 22 samples were tested from the district during this period. Increased efforts to laboratory confirm cases could improve confidence in clinical diagnosis. In general, we believe that our case definition for probable rabies was conservative and likely underestimates the true proportion of rabies exposures (see Section 2, *case definitions*). Moreover, we may have underestimated rabies exposures and overestimated reporting as investigations were initiated only for patients reporting to the ARMC. We likely missed individuals that reported only to peripheral clinics or that did not report at all (that were not linked to other ARMC patients). Bites by vaccinated dogs also appear to be disproportionaly represented in the ARMC as unpublished data from a recent vaccination campaign suggests much lower dog vaccination coverage before the campaign was implemented (5% pre-April 2018, M. Rajeev unpublished data). During our study, only six patients bitten in the Moramanga District reported directly to IPM without referral, the nearest other ARMC, suggesting that most bite victims in the district that seek PEP are captured at the Moramanga ARMC.

We make several further simplifying assumptions in our estimations of rabies burden in the Moramanga District and our extrapolations to Madagascar. We did not incorporate risks due to incomplete or delayed PEP or for severe exposures that did not receive RIG. Since no deaths were reported from patients who received delayed/incomplete PEP, PEP completion was high, and severe exposures (i.e. deep wounds) uncommon, we believe this will not have introduced major bias. We did not account for vaccine availability and assumed that all patients that reported to the ARMC received PEP. Although the clinic did not experience PEP shortages during our study, in March 2018 the entire country experienced a stockout, with no vaccine available at the Mora-manga ARMC for two weeks. We also assumed uniform rabies incidence and reporting across Madagascar. Given that only 31 of 114 districts have an ARMC, this likely underestimates the burden and overestimates deaths averted. Data from other districts on bite incidence, rabies exposures, health seeking behavior, and PEP adherence and availability would improve our estimates and understanding of rabies risk across Madagascar.

### 4.3 Wider context

The animal and patient characteristics described in our study are similar to most other rabies endemic settings, with domestic dogs responsible for the majority of animal bites and rabies cases [19–21], rabies exposures disproportionately affecting children under 15 years of age [12,22], patient demographics skewed male [15,23,24], and high risk exposures (i.e. deep wounds or bites to the head or neck) generally rare [25]. Unlike in some other rabies endemic countries, the majority of patients reported washing the wound with soap and water, which can greatly reduce risk of transmission [26–29]. Our estimates of incidence of bite patients, rabies exposures, and human rabies deaths were similar to those from a wide range of endemic settings [19,21,22,29]. This is the first estimate of rabies burden in Madagascar based on data specific to the country and is in line with the previous estimate of burden for Madagascar using data from sub-Saharan Africa [1].

A higher proportion of suspect exposures sought (~85%) and completed PEP (~90%) compared to other regions with endemic rabies, where PEP is only available at a high cost to patients and a lower proportion of rabies exposed persons receive PEP [21,22,24,30,31]. Few other studies have described health-seeking behavior and PEP adherence in settings where PEP is free; however, in both Bhutan and Phnom Phen in Cambodia where PEP is provided at no charge to patients, approximately 80–90% receive and complete PEP [15,32]. Regardless of whether PEP is free, costs to patients (in the case of free PEP, indirect costs) and limited geographical access seem to present the greatest barriers [17,22,33]. In addition, awareness on the part of both patients and clinicians responsible for referrals also contribute to bite victims not receiving PEP [34].

We were able to determine the rabies status for animals we investigated to a comparable level as that reported in similar studies in Haiti and Tanzania [20,35]. Overall, approximately 20% of animal bites were determined to be likely rabies exposures compared to 13% in Haiti [20], 73% in Ethiopia [21], and 62% in Tanzania [22]. This variation may be due to differences in dog vaccination, as well as higher health seeking of people bitten by non-rabid animals in free settings (higher levels of dog vaccination coverage in Haiti and Tanzania; more costly PEP in Ethiopia and Tanzania). Our results suggest that implementing recently developed integrated bite case management programs which use risk assessments to prioritize PEP administration [17,34,36] and using bite patients as sentinels for rabies surveillance [35] are feasible and effective options to better manage PEP and improve surveillance in Madagascar, especially as control in the dog population is implemented.

### 4.4 Conclusions & recommendations

Our findings show that canine rabies is responsible for a high incidence of human rabies exposures and preventable rabies deaths in Madagascar, and accounts for a large proportion of the demand for PEP. Given current successful ID administration of PEP and vial sharing practices, adoption of the latest WHO recommendations for PEP administration using the abridged 1-week ID regimen could be implemented immediately in Madagascar to reduce PEP costs. Shifting away from control strategies of reactive culling to mass dog vaccination would further reduce both the high costs of PEP and the burden of human rabies. Increasing access to PEP and awareness for its need could also greatly reduce the burden of human rabies, especially given its limited availability within Madagascar. Nonetheless, the fact that where PEP is available, it is provided to patients for free, appears to result in relatively high health seeking and adherence in comparison to other low-income settings. In general, more judicious use of PEP may be warranted as access is expanded and vaccine use increases. Particularly, if mass dog vaccination is implemented and risk of rabies exposures decrease, integrated bite case management [36] could be used to further reduce PEP demand while enhancing surveillance of animal cases and identification of exposed persons [20,35]. However, this would require improved integration of activities and coordination between the health and veterinary sectors. Given the push to eliminate deaths due to human rabies [4], our results demonstrate that investing in rabies control as a public good through providing free PEP can prevent needless human deaths, and in combination with mass dog vaccination has the potential to greatly reduce and eventually eliminate rabies from Madagascar.

## Figures and Tables

**Table 1:** Characteristics of biting animals as recorded from follow-up investigations.

		Confirmed (%)	Probable (%)	Unknown (%)	Non-case (%)
Total		19	68	108	195
Species	Cat	3 (15.8)	2 (2.9)	9 (8.3)	21 (10.8)
	Dog	15 (78.9)	61 (89.7)	91 (84.3)	173 (88.7)
	Bovine	1 (5.3)	5 (7.4)	0 (0)	1 (0.5)
	Rodent	0(0)	0 (0)	6 (5.6)	0 (0)
	Owned animal	16 (84.2)	42 (61.8)	30 (27.8)	189 (96.9)
	Vaccinated	0 (0)	0 (0)	9 (8.3)	57 (29.2)
	Veterinary observation	3 (15.8)	5 (7.4)	4 (3.7)	68 (34.9)
Outcome	Alive	0 (0)	0 (0)	14(13)	186 (95.4)
	Disappeared or unknown	0 (0)	17 (25)	81 (75)	0 (0)
	Died due to disease	4(21.1)	19 (27.9)	0 (0)	1 (0.5)
	Killed after biting a person/animal	14 (73.7)	23 (33.8)	4 (3.7)	2(1)
	Other cause of death	0 (0)	9 (13.2)	2 (1.9)	6(3.1)
Clinical signs	Bit multiple people	11 (57.9)	26 (38.2)	0 (0)	10(5.1)
	Bit other animals	5 (26.3)	10 (14.7)	1 (0.9)	0 (0)
	Observed source of infection(i.e. signs of previous bite/observed bite)	4(21.1)	5 (7.4)	2 (1.9)	2(1)
	Unprovoked aggression	12 (63.2)	47 (69.1)	41 (38)	33 (16.9)
	Excess salivation	6 (31.6)	14 (20.6)	3 (2.8)	2 (1)
	Hydrophobia	1 (5.3)	1 (1.5)	0(0)	0 (0)
	Lethargy	2 (10.5)	7 (10.3)	0 (0)	0 (0)
	Paralysis	1 (5.3)	5 (7.4)	0 (0)	1 (0.5)
	Vocalization	3 (15.8)	4 (5.9)	0 (0)	1 (0.5)
	Restlessness	3 (15.8)	0(0)	0 (0)	0 (0)
	Hypersexuality	0 (0)	0 (0)	0 (0)	0 (0)
	Running no reason	4 (21.1)	7 (10.3)	0 (0)	0 (0)
	Strange movement	2 (10.5)	8 (11.8)	1 (0.9)	1 (0.5)
Provoked bite*		5 (26.3)	10 (14.7)	24 (22.2)	57 (29.2)
Average number of animals bitten		0.313	0.236	0.019	0
Average number of humans bitten		2.06	1.73	1	1.05

* With at least one indication of provocation (i.e. hitting or kicking the animal, interaction with food or object, playing or running, entering the house of the owner with a guard dog, history of habitual aggression).

**Table 2:** Characteristics of all patients reporting for PEP and additional bite victims identified through contact tracing, including the type of exposure and health seeking behaviour.

		Confirmed (%)	Probable (%)	Unknown (%)	Non-exposure (%)	Contact (%)
**Total**		**35**	**85**	**425**	**202**	**197**
Average age		23.5	23.8	23.7	25.5	30.7
Male		27 (77.1)	50 (58.8)	250 (58.8)	106 (52.5)	125 (63.5)
15 yrs or younger		19 (54.3)	39 (45.9)	189 (44.5)	84 (41.6)	46 (23.4)
Unreported		2 (5.7)	17 (20.0)	1 (0.2)	7 (3.5)	–
**Total reported**		**33**	**68**	**424**	**195**	**197**
Completing at least 3 doses		29 (87.9)	63 (92.6)	383 (90.3)	170 (87.2)	178 (90.4)
Completing at least 4 doses		29 (87.9)	56 (82.4)	316 (74.5)	129 (66.2)	157 (79.7)
Completing more than 4 doses		1 (3)	3 (4.4)	8 (1.9)	3 (1.5)	1 (0.5)
Average delay between exposure and reporting (days)		1.5	2.8	2.6	1.8	NA
Reported within 2 days of bite		28 (84.8)	48 (70.6)	324 (76.4)	159 (81.5)	–
**Interviewed**		**35 (1 0 0)**	**82 (96.5)**	**111 (26.1)**	**171 (84.7)**	**–**
Reported to peripheral clinic before reporting to the ARMC		0 (0)	6 (7.3)	15 (13.5)	21 (12.3)	–
Reported to a peripheral clinic only (unreported to ARMC)		0 (0)	7 (8.5)	0 (0)	3 (1.8)	–
Reported to any other hospital		0 (0)	13 (15.9)	15 (13.5)	24 (14)	–
Wound location**	Legs	8 (22.9)	31 (37.8)	51 (45.9)	79 (46.2)	–
	Feet	4 (11.4)	15 (18.3)	26 (23.4)	26 (15.2)	–
	Arms	9 (25.7)	5 (6.1)	6 (5.4)	14 (8.2)	–
	Hands	8 (22.9)	23 (28)	23 (20.7)	24 (14)	–
	Upper body	5 (14.3)	4 (4.9)	7 (6.3)	24 (14)	–
	Head or neck	2 (5.7)	4 (4.9)	1 (0.9)	6 (3.5)	–
Wound type**	Skin broken	21 (60)	57 (69.5)	92 (82.9)	126(73.7)	–
	Superficial	25 (71.4)	59 (72)	94 (84.7)	134 (78.4)	–
	Deep	2 (5.7)	5 (6.1)	6 (5.4)	10(5.8)	–
	Scratch	8 (22.9)	15 (18.3)	18 (16.2)	32 (18.7)	–
	Bite	29 (82.9)	64 (78)	91 (82)	144 (84.2)	–
	Multiple	1 (2.9)	1 (1.2)	1 (0.9)	3 (1.8)	–
	Over clothes	7(20)	9(11)	31 (27.9)	45 (26.3)	–
Washed wound		28 (80)	62 (75.6)	96 (86.5)	139 (81.3)	–

Bold rows are denominators for subsequent rows.

** Categories are not mutually exclusive and were assigned as they applied to each bite victim.

**Table 3:** Details of the human deaths in the district during the study period.

Case	Age	Sex	Type of exposure	Biting animal	Health-seeking and wound response	Time between bite and death
Confirmed	3	F	Superficial scratch to the face	Owned dog, killed after biting	Did not report to the CTAR or any other hospitals; did not wash wound, but applied tambavy (a local plant).	~2 months
Suspected	67	M	Bite, no details on location	Owned dog, disappeared after the bite	No details but did not report for PEP.	~1 month
Suspected	61	M	Superficial bite to the hands	Owned dog, killed after biting	Reported to peripheral clinic and was referred to the CTAR, but did not report for PEP; washed wound and applied oil.	~2 months
Suspected	45	M	Deep bite to the hands	Unknown dog, disappeared after the bite	Reported to peripheral clinic and was referred to the CTAR, but did not report for PEP; washed wound.	~1 year

**Table 4:** Parameters for decision tree model (note that exposures exclude contacts with probable cases).

Parameter	Value	Description
Overall bite incidence per 100,000 people	189	12 × average of monthly bites (both unreported and reported) between Aug and Dec 2017, when systematic triage was in place
Proportion of overall bites due to rabid animals, p_rabid_	0.22-0.58	The average monthly proportion of probable/confirmed exposures only (lower limit) or probable/confirmed AND unknown exposures (upper limit) between Aug and Dec 2017, when systematic triage was in place.
Proportion of rabies exposures that seek PEP, p_report_	0.84	The proportion of probable/confirmed exposures which reported to the ARMC
Proportion of rabies exposures that result in infection in the absence of PEP, p_infect_	0.164	Changalucha et al. 2018 (submitted) [12]
Moramanga population	328,000	Midpoint between World Pop 2015 and 2020 UN adjusted population projections [8]
Madagascar population	26,017,000	Midpoint between World Pop 2015 and 2020 UN adjusted population projections [8]

## Data Availability

Anonymized data are available on request and pending approval of the Ministry of Public Health and IPM.
